# Repertory of the Back

**Published:** 1898-06

**Authors:** E. H. Wilsey

**Affiliations:** Parkersburg, W. Va.


### REPERTORY OF THE BACK.

E. H. Wilsey, M. D., Parkersburg, W. Va.

(Continued from May number, page 203.)

### SPINE.

**Lying,** pains in spine, better by lying on something hard, | Nat-m.

— spinal irritation, better lying flat on back, with firm pressure, | Nat-m.

**Lower,** pain in lower part of spine and trembling of limbs, | Coccul.

**Meningitis,** convulsions, with jerking of muscles in spinal meningitis, | Hyos.

— inflammation of meninges after abuse of Merc. or secondary syphilis, | Kali-iod.

— chronic spinal meningitis, with sudden loss of flesh, Plumb.

— spinal meningitis during scarlatina or measles, eruption does not develop, | Dulc.

— cerebro-spinal meningitis in the tetanic stage, | Physos.

— softening of the spinal cord, congestion, or irritation, spinal meningitis, myelitis, or spinal paralysis, Crotal.

**Middle,** drawing pain in middle of spine, with drawing pains opposite to it on back part of stomach, Stram.

— pain in middle of spine, Sambucus.

— pinching pain in middle of spine, Phos-ac.

— pains in middle of spine when sitting, | Arn.

**Morning,** pain, as if bruised, in the whole spinal column, in the morning on waking, while lying on his back, Mag-m.

— severe aching down spine, worse from friction, passing off after rising, mornings, Lyc-vir.

SPINE.

**Morning,** pain in middle of spine in morning, with general bruised feeling, worse upper limbs, Tabac.
— painful stiffness in morning on rising, with indolence and heaviness of legs, Calc-c.
— pain in spine in morning, so severe he could not move a limb, Aur-fol.
**Morbid** feeling in different parts of spine, Tabac.
**Motion,** tearing downward on the whole spine in rest and in motion, Mang.
— painful feeling in spine on motion, Lob-c.
**Myelitis** (inflammation) **I** Acon., Amm-c., Ant-c., **I** Arn., Ars., Bar-m., **I** Bell., **I** Benz-ac., Bry., **I** Calab., **I** Calc., Carbo-v., Caust., Cham., Cic., Cina, China, Coccul., Coff., **I** Colch., Con., Crotal., **I** Dulc., Euph., Hep., Hyos., **I** Hyper., **I** Ign., Iod., Kali-iod., **I** Lach., Lyc., **I** Merc., Nat-m., Nit-ac., Nux-m., **I** Nux-v., **I** Op., Oxal-ac., **I** Phos., Phos-ac., **I** Pic-ac., Puls., **I** Rhus, Ruta, Sars., Sec., Sep., Sil., **I** Sul., Sul-ac., **I** Ver.
— diffusa, **I** Secale.
— after taking cold during menstruation, **I** Dulc.
— with paraplegia of extremities, of bladder and anus, with a tendency to twitching and shocks, **I** Merc.
— and early stages of locomotor ataxia, especially when occurring from exposure to cold or from sexual excess, **I** Nux.
— painful, paralytic weakness in upper and lower limbs, **I** Verat.
— with numbness and insensibility of extremities, **I** Phos.
— after sexual excesses, or getting wet, **I** Phos.
— spinal membranes inflamed, even myelitis from getting wet or sleeping on damp ground, **II** Rhus.
— with diabetes, Phos-ac.
— anterior horns inflamed, early stage, **I** Gels.
— with meningitis after injury, **I** Hyos.
— in meningitis after injury or checked sweat, **I** Acon.
— worse at night and from cold, while lying down, and from washing, **I** Dulc.

SPINE.

**Myelitis,** osteomyelitis, I Merc-c., I Phos.

— causes prolapsus uteri, I Sil.

— syphilitic, I Kali-iod., I Merc., I Nit-ac., I Phyto., Sang., Thuja.

— perimyelitis, Sep.

**Neuralgia** of spine, I Ars., Diad., I Gels., I Coloc., Ran-b., I Glon., I Lach.

— in top of spine, extending to vertex, I Ars.

— pressure on spinous process of first three cervical vertebræ, worse or brings on neuralgic attacks, I Coloc.

— worse morning and evening, and after eating (rheumatic neuralgia after unusual exposure), Phos.

**Numb,** tired pains up and down spine and in head, I Curare.

**Numbness** and formication along spine and into extremities, I Nux-v.

— myelitis, with numbness and insensibility of extremities, I Phos.

— in back radiating from spine, Populus-c.

— spinal irritation, numbness in lower extremities, with great prostration, Zinc.

**Oppression** of spine during sweat, Sep.

**Onanists,** feeling of lightness in body from spinal exhaustion in onanists and hysterical subjects, I Gels.

**Pain,** sensation as if atlas and axis had been pried apart; on rising to feet it changed to a sharp pain, Curare.

— in region of seventh cervical vertebra after a walk, with drawing in nape, Curare.

— sore pain the whole extent of spine from below upward, Eup-pur.

— in neck like those of cerebro-spinal congestion, II Gels.

— from spine to head and shoulders, I Gels.

— in spine as if beaten and like lumbago, I Ruta.

— in last dorsal vertebra, I Zinc.

— in spine when walking, then drawing pressure as if bruised; better by pressure, Ver-a.

SPINE.

**Pain,** severe in lower part of spine, Lyss.
— severe pains in spine, worse from pressure, | Merc.
— spine sore and painful, | Merc-i-rub.
— pulsating near middle of spine, Mez.
— down spine, with twitching, Morphinum.
— in middle of spine in morning, with general bruised feeling, worse upper limbs, Tabac.
— severe bruised pain in whole spinal column at night, Mag-m.
— all down spine and close to it on both sides, Calabar.
— in spine, Calad.
— in spine on bending backward, Calc-c.
— in fifth, sixth and seventh cervical vertebræ, worse moving head; better from pressing hand thereon, | Camph.
— at base of spine, extending along down sciatic nerve and in hip-joint, Can-s.
— near spine, right side, where scapula ends, Alum.
— as if not able to carry body, | Arn.
— in middle of spine, when sitting, | Arn.
— much pain in spine at nape of neck and sacrum, especially worse on pressure, | Angustura.
— cutting pains extending in a circle from spine to abdomen, | Acon.
— all along the spine; stiff neck, | Cedron.
— in spine, between shoulders, | Chel.
— in spine, painful on pressure at all stages of fever paroxysm, || Chin-sul.
— down entire spine, after constriction in chest, shivering downward, | Glon.
— great pain in spine after a fall from a hammock, growing daily more severe, | Hypericum.
— in spine, spreading to kidneys, Lil-tig.
— constant in spine, sometimes worse in lumbar region, with great heat and burning, | Kalmia.
— on turning or moving head, pain in spine of neck into brain, | Lach.

SPINE.

**Pain,** pressure on spine of neck sends pain to brain, **I** Lach.
— extends to extreme end of spine, Ustilago.
— as if in the spinal marrow extending through coccyx, Lactu-v.
— very great pain behind and above right hip on a small spot near spine; worse walking, had to sit down, Ars-h.
— in lower part of spine, Aur-m.
— in lumbar region of spine, followed by apoplexy and paralysis, **I** Bary-carb.
— extends down spine, **I** Coccul.
— sensitiveness of vertebræ to touch, but cannot locate pain, **I** Coccul.
— in spine, better by lying on something hard, **I** **Nat-m.**
— between shoulders and along spine, **I** Nux-m.
— periodically returning insupportable pains in spine, preventing walking, **I** Phos.
— and stiffness down spine, with inclination to bend forward, as if had to sit up straight, Physos.
— down part of spine, across hips and down thighs, with difficult urination, Sarsa.
— in spine, preventing sleep, Con.
— in third cervical vertebra, **I** Fluor-ac.
— in cervical spine, in influenza, **I** Cepa.
— in spine, attending curvature, Æsc-h., **II** Sil.
— in spine, with depression, **I** Naja.
— in spine, worse stooping, **I** Agari.
— in spine, streaking up and down, **I** Phyto.
— in spine, like a weight, worse on lifting, **I** China.
— in fifth and sixth vertebræ, Aspar.
— in spine, cannot walk erect, must stoop, Can-in.
— in spine, as if tired, in lumbar vertebra in morning, Kreos.
— sudden in fourth and fifth dorsal vertebræ, to right side of chest and shoulders, Brach.
— in middle of spine on swallowing, as if a sharp crust was sticking in an inward direction, **I** Caust.

### SPINE.

**Paralysis** of spinal cord, ǀ Con., Crotal., Eucal., Sec., ǀ Phos., ǀ Sil., ǀ Nat-m., Helleb., ǀ Kali-ph., ǀ Rhus, ǀ Ars.
— of limbs from spinal softening, Stram.
— progressive paralysis of spine, with partial contraction of affected muscles, anæsthesia and increased heat, ǀ Phos.
— congested state of paralysis of spinal cord, with tetanic spasms, ǀ Physos.
— partial paralysis from weakness of spine, ǀ Nat-m.
— of lower third of spine, ǀ Ars.
— myelitis: painful paralytic weakness in upper and lower limbs, ǀ Ver-a.
— of spine, with tetanus, Kali-nit.
— spinal irritation, paralytic symptoms, cold feet and constipation, ǀǀ Sil.
— of spinal cord, from exhaustion, ǀ Nux-v.
— exhaustion from drainings affecting nerve-centres, ǀ Kali-p.
— of spine in infants, ǀ Rhus.
— of spine from exhaustion by sexual excesses, ǀ Kali-br.
— of lower third of spine, ǀ Ars.
— of spine from inflammation, Oxal-ac.
— of spine from decay of anterior portion, Mang.
**Paraphlegia,** ǀ Gels.
**Pinching** near lowest portion of spine, ǀ Carbo-v.
— pain in middle of spine, Phos-ac.
**Plug,** pain near spine, as if a plug were forced inward two inches below left scapula on stooping, Prunus.
— pain, as from a plug in right side, near middle of spine, and on pressure pain as in a wound, Plat.
— a feeling as of a plug sticking in spine, and any motion causes pain as if plug was sticking still further into body, Anac.
**Potts' curvature,** ǀ Sul., ǀ Calc-c., ǀ Calc-ph.
**Pollution,** spine affected after pollutions; excessive weakness shows itself in the legs, ǀ Sabad.
**Pressing** sensation passing up and down through spine while sitting erect, Spong.

SPINE.

**Pressure,** great sensitiveness of cervical vertebra to pressure, | Arn.
— spine tender on pressure, Plant.
— pain as from a plug in right side near middle of spine, and on pressure pain as in a wound, Plat.
— drawing, very acute pain in right side of spine opposite liver, especially on inspiration, Ruta.
— along spine while walking in open air, disappearing while sitting and standing, Mur-ac.
— pinching, pressing near lowest portion of spine, | Carbo-v.
— spine painful on pressure at all stages of fever paroxysm, || Chin-s.
— sensitiveness of last cervical and first dorsal vertebræ to pressure, || Chin-sul.
— aching soreness in all the vertebræ ; worse by motion and by pressure on spinous processes, | Chel.
—dyspnœa caused by pressure on upper three dorsal vertebræ, | Chin-s.
— of finger between vertebræ causes patient to wince, | Physos.
— spinal irritation, better by lying flat on back with firm pressure, | Nat-m.
— dorsal region of spine tender to pressure, Plumb.
— from spine to stomach before breakfast, Nat-phos.
— and stitches in region of spine, Nit-s-dulc.
— great tenderness on pressure over posterior spinous processes of all cervical and first four dorsal vertebræ, | Coloc.
— on spinous processes of first three cervical vertebræ worse or brings on neuralgic attacks, | Coloc.
— drawing pressure between right scapula and spine, Bell.
— on spine of neck sends pain to brain, | Lach.
— in spine during deglutition, Kali-c.
— spine sensitive to slightest pressure, which causes outcries and raving, Stram.
— severe spinal pains, worse from pressure, | Merc.

SPINE.

**Pressure,** oversensitiveness of spine to touch or upon pressure, | Nat-m., Tell., | Sep.
— in right side, near spine, Zinc.
— spinal irritation, great sensitiveness between vertebræ, sits sideways in a chair to avoid pressure against spine, | Therid.
— abortive eructation, with pressure in middle of spine, Zinc.
— on spine above lumbar region, with rheumatic drawing in neck, Sep.
— in spinal process of seventh dorsal vertebra, Ginseng.
**Pressive** pain in spine between the scapulæ, with short breath, worse on respiring, with pain in spinal vertebra on being touched, Calc-c.
— squeezing, pressive pain beside the lowest part of spine, Carbo-v.
— stitches along spine, mostly in sacrum, with dyspnœa, Tarax.
**Pricks,** violent, as from needles in middle of spine, almost causing him to cry out on taking a walk in open air, somewhat better by standing, Calc-c.
**Pricking** up and down spine, Jug-cin.
— weakness in dorsal region of spine, with intermittent prickings, Raphanus.
**Prostration,** spinal irritation, numbness in lower extremities, with great prostration, | Zinc.
**Pulsation** in the spine when sitting, Thuja.
— a dull, pulsating pain close beside the middle of spine, Mez.
— extending from neck down spine to lumbar region in P. M. while sitting, Curare.
— painful pulsation in spinal canal, Agari.
**Purple,** discolored band two inches wide along each side of spine, Chloral.
**Raised,** cannot bear to be raised up, and cannot raise herself in bed because of severe pain in spine, very little sleep, | Lach.
**Rheumatism** of spine, | Phyto., | Dulc.
— of spine, pain passes up neck to occiput, | Phyto.
— of lower cervical vertebra, | Sil.

SPINE.

**Rheumatism,** multiple sclerosis from fright and rheumatism, Tarant.
— in upper cervical vertebra, with stiffness of neck, **I** Cal-c.
**Rheumatic** affection of spinal cord, **I** Dulc.
— spinal irritation, Caustic., Bar-carb.
— tensive pain in spine, Zinc.
**Rigidity** of spine, **II** Bell.
— of spine great, **I** Lob-c.
— of spinal column in periodic attacks, accompanied by pains through whole chest, Cepa.
**Rising,** violent pains in spine, as from sudden rising after long stooping, **I** Arn.
— painful stiffness of spine in morning on rising, with indolence and heaviness of legs, Calc-c.
**Rumbling** inside along spine, Sul.
**Sclerosis,** lateral, **I** Nux-v., Crotal.
— posterior, Plumb., **I** Sil., Pic-ac.
— multiple, especially in beginning, with gastralgic attacks, vertigo, etc., **I** Nux-v.
— multiple, Arg-n., Bar-c., Bell., **I** Calab., **I** Caustic., Crotal. Gels., Ign., **I** Nux-v., Oxal-ac., **II** Phos., **I** Pic-ac., **I** Plumb., **I** Rhus, **I** Sil., Tarant.
— multiple, from fright and rheumatism, Tarant.
**Sensitive,** cervical vertebra very sensitive to touch, **I** Hyper.

---

THE REPERTORY OF THE BACK.—In answer to the numerous inquiries concerning this repertory the Editor desires to state that it is only about half completed. There are several sections to be added, such as Lumbar, Sacrum, etc. The whole is being reprinted that a separate book may be made of it, when it can be procured in this office.—ED.

